# Supplementary material for: Asymmetric Synthesis of Quaternary Hydantoins via a Palladium-Catalyzed Aza-Heck Cyclization
Source: J Am Chem Soc. 2025 Nov 14;147(49):44692–8. doi: 10.1021/jacs.5c16022 (PMC12703750; doi:10.1021/jacs.5c16022)
Supplement: Supplementary file 2 [file ja5c16022_si_002.zip › All NMR FID Files/S35/S35_AllNMR/2/pdata/1/email_EM04-153-dry_2_1.pdf]

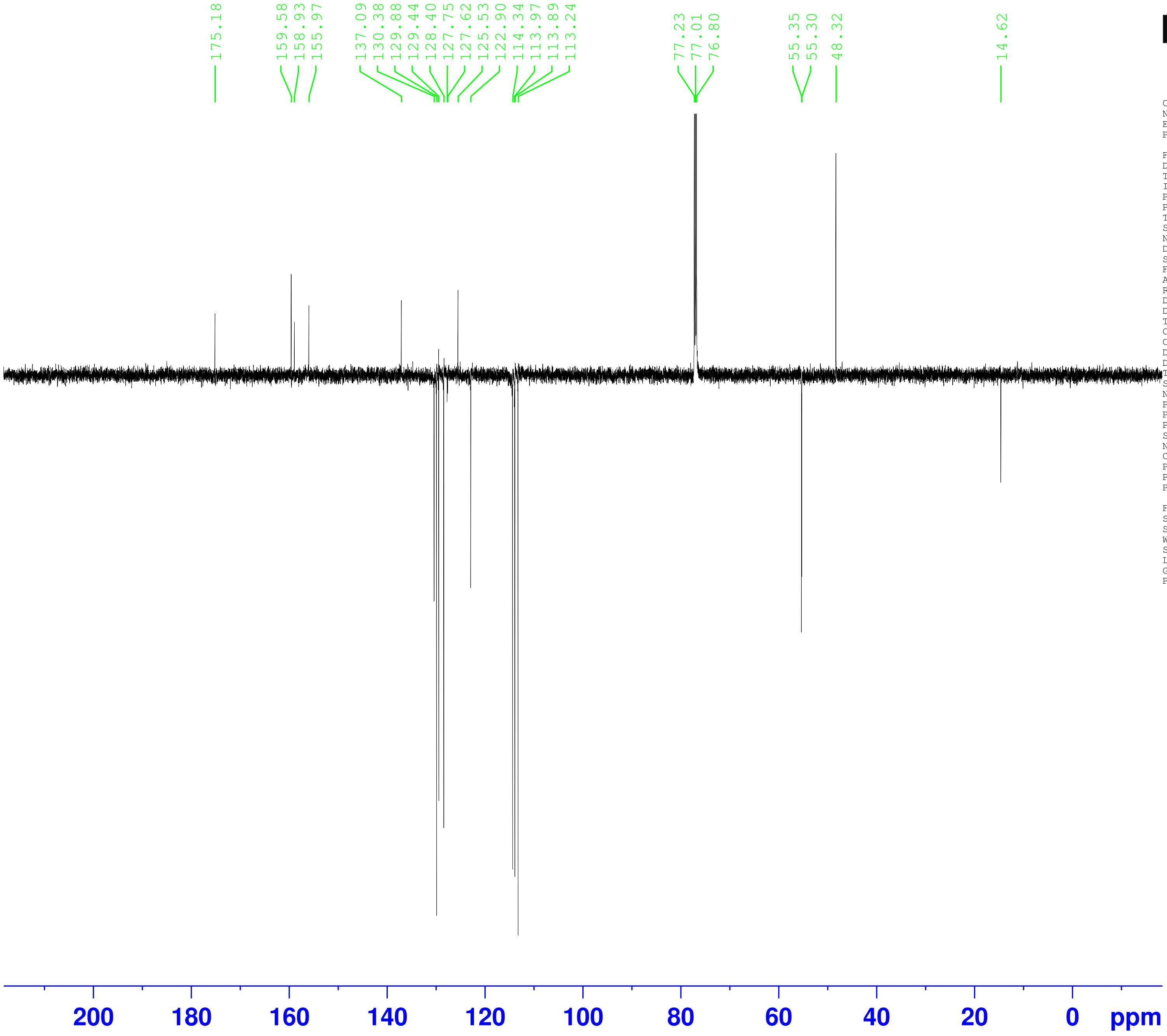

Current Data Parameters

NAME	EM04-153-dry
EXPNO	2
PROCNO	1

F2 - Acquisition Parameters

Date_	20231007
Time	12.43 h
INSTRUM	Avance Neo 600 at University of Delaware
PROBHD	Z185170_0001 (
PULPROG	jmod
TD	65536
SOLVENT	CDCl3
NS	2048
DS	4
SWH	35714.286 Hz
FIDRES	1.089913 Hz
AQ	0.9175040 sec
RG	101
DW	14.000 usec
DE	19.25 usec
TE	299.1 K
CNST2	145.0000000
CNST11	1.0000000
D1	2.00000000 sec
D20	0.00689655 sec
TD0	1
SFO1	150.9178988 MHz
NUC1	13C
P1	11.78 usec
P2	23.56 usec
PLW1	106.00000000 W
SFO2	600.1324005 MHz
NUC2	1H
CPDPRG[2	waltz65
PCPD2	70.00 usec
PLW2	6.90000010 W
PLW12	0.09034800 W

F2 - Processing parameters

SI	32768
SF	150.9028085 MHz
WDW	EM
SSB	0
LB	1.00 Hz
GB	0
PC	1.40
